# Use of Chlorogenic Acid against Diabetes Mellitus and Its Complications

**DOI:** 10.1155/2020/9680508

**Published:** 2020-05-28

**Authors:** Yongwang Yan, Xu Zhou, Kangxiao Guo, Feng Zhou, Hongqi Yang

**Affiliations:** ^1^College of Bioscience and Biotechnology, Hunan Agricultural University, Changsha, China; ^2^Pharmaceutical College, Changsha Health Vocational College, Changsha, China; ^3^Department of Gastroenterology, Changsha Hospital of Traditional Chinese Medicine, Changsha, China

## Abstract

Chlorogenic acid (CA) is a phenolic compound commonly found in human plant-based diets. CA is the main component of many traditional Chinese medicine preparations, and in recent years, it has been found to have hypoglycemic, hypolipidemic, anti-inflammatory, antioxidant, and other pharmacological properties. Specifically, CA relieves the effects of, and prevents, diabetes mellitus (DM). In addition, CA is also beneficial against complications arising from DM, such as diabetic nephropathy (DN), diabetic retinopathy (DR), and diabetic peripheral neuropathy (DPN). Herein, we review the use of CA in the prevention and treatment of DM and its complications, providing a background for further research and medical uses.

## 1. Introduction

Chlorogenic acid (CA), also called 5-caffeoylquinic acid (5-CQA) [1], belongs to the hydroxycinnamic acid family, and is formed by caffeic acid and quinic acid [2, 3]. CA is produced in plants in the shikimic acid pathway during aerobic respiration. This compound is an ingredient, not only in foods, but also in traditional Chinese medicine preparations [4, 5]. In the latter, it has been found to exert hypoglycemic [6, 7], hypolipidemic [8], antibacterial [9], antioxidant [10], and anti-inflammatory [11] effects. In this context, the hypoglycemic and hypolipidemic effects of CA have attracted attention, specifically in their possible application in the prevention and treatment of diabetes mellitus (DM) [12–14]. DM is a metabolic disease caused by abnormal insulin function and characterized by hyperglycemia. Its main subtypes are (1) type 1, with absolute insulin deficiency, and (2) type 2 DM, or noninsulin-dependent DM, with relative insulin deficiency and resistance.

According to estimates of the World Health Organization, DM affects 366 million patients worldwide, a number expected to grow to 500 million by 2030 [15]. DM is a high-risk factor for stroke, heart disease, and kidney disease, thus, seriously affecting the quality of life and severely restricting social and economic development [16]. Despite many studies reported the use of CA against DM and its complications, these studies have not been systematic, and information is scattered in the literature. The present review aims at organizing this information for further research work and medical use.

## 2. Use of CA to Prevent and Treat DM

### 2.1. Effect of CA on Glucose Metabolism

Persistent hyperglycemia is the overriding feature of DM. At the time of the onset of DM, islet *β* cells continuously and excessively secrete insulin to reduce blood glucose, which eventually causes islet *β* cell injury and aggravates hyperglycemia. Under persistent hyperglycemia, glucose toxicity causes DM chronic complications [16]. CA has been shown to reduce fasting blood glucose; for example, when 15 patients with impaired glucose tolerance were exposed to 400 mg CA, administered three times a day for 12 weeks, in a randomized, double-blind, placebo-controlled clinical trial [12]. In other clinical trials, CA-containing green coffee bean extract was used to reduce fasting blood glucose in 21 patients with metabolic disease, when the extract was administered in 400 mg capsules given twice a day for a total of 8 weeks [14]. Further, blood glucose in mice on a high-fat diet treated with green coffee bean extract, which is majorly composed of CA, was significantly lower than in a control group, when the extract reached 100 mg/kg body weight, after six weeks [17]. In another study, rats with type 2 DM were treated with CA-containing mulberry leaf extract, rutin, or isoquercitrin for 11 days. While mulberry leaf extract, CA, and rutin markedly reduced blood glucose in the treated rats, isoquercitrin had no obvious hypoglycemic effect, suggesting that over 50% of the hypoglycemic effect observed in mulberry leaf extract could be attributed to CA and rutin [18]. Also, lower fasting blood glucose and a raised muscle glycogen were found in laboratory db/db mice when administered CA by gavage at a dose of 80 mg/kg/day for 12 weeks [19]. During a study of the effect of CA on postprandial blood glucose content, Tunnicliffe et al. [20] found that blood glucose in rats treated with CA for 60 min after a meal was markedly lower than in rats treated with placebo. Finally, lower than normal blood glucose was reported when streptozotocin-nicotinamide-induced DM rats were treated with CA at a dose of 5 mg/kg/day for 45 days, with levels in treated and control rats of 105.2 and 282.28 mg/dL, respectively [21].

### 2.2. Effect of CA on Lipid Contents

Dysfunctional lipid metabolism is a well-known high-risk factor in DM [16], and several reports have highlighted the effect of CA in improving lipid metabolism. In Wistar rats exposed to a high-glucose and high-fat diet, CA improved lipid metabolism, reduced weight gain, liver weight, mesenteric and epididymal fat weight, contents of liver cholesterol, triglyceride, free fatty acids, and plasma free fatty acids [13]. Similar observations were reported in other animal experiments regarding weight gain, liver weight, and plasma free fatty acids [17], and in mice exposed to a high-fat diet, green coffee bean extract which is majorly composed of CA reduced plasma triglycerides, low-density lipoproteins, and high-density lipoproteins. Ong et al. [22] reported that db/db mice treated with CA at a dose of 250 mg/kg/day for 14 days showed significantly reduced levels of total cholesterol, triglycerides, and free fatty acids in plasma, relative to a control group. Moreover, liver histomorphology showed that CA inhibited the formation of fat particles in the hepatocytes of treated mice.

In SD rats exposed to a high-glucose and high-fat diet, CA dramatically reduced total cholesterol, low-density lipoprotein, high-density lipoprotein in plasma, and liver lipid content [23], but had no effect on triglycerides. This is in contrast with another report [19] that claimed just the opposite; an effect of CA on triglycerides, but no reduction of free fatty acids and total cholesterol in blood, liver, or muscle.

In a study that assessed the effect of CA and tetrahydrocurcumin on blood lipids in DM rats, CA significantly reduced contents of cholesterol, triglycerides, free fatty acids, high-density lipoproteins, low-density lipoproteins, very low-density lipoproteins in plasma, and lipids in liver and kidney [21]. Finally, in golden hamsters fed a high-fat diet, CA inhibited weight gain by reducing the content of visceral fat, plasma triglycerides, total cholesterol, free fatty acids, high-density lipoprotein, and low-density lipoprotein, as well as triglyceride and total cholesterol in the liver and free fatty acid in muscles [24].

### 2.3. Effects of CA on Insulin Secretion and Resistance

CA has been reported to relieve insulin resistance which is the direct cause of DM [25, 26]. In a clinical test [12], a decline in fasting blood glucose and insulin secretion of patients treated with CA for 12 weeks, suggested that CA can ameliorate insulin resistance and enhance insulin sensitivity. However, in another clinical experiment, CA did not increase the secretion of glucagon-like peptide-1 and glucose-dependent insulin-stimulating hormone [27]. Experiments performed in *β* cells pretreated with CA have shown that the secretion of insulin was increased after culture in media with 4 mM or 10 mM of glucose [28]. In another experiment with the INS-1E insulin-secreting cell line and rat islets of Langerhans, stimulation of insulin secretion was observed after treatment with 50 *μ*g/mL CA; the effect was close to that caused by 5 mM of glucose, whereas in 8.3 mM of glucose, CA significantly increased the secretion of insulin [29]. Rats exposed to a high-fat diet and administered 50 mg/kg CA for 20 weeks showed an increase in insulin secretion and an improvement in insulin resistance [30]. Also, CA treatment of both high-fat diet-induced obese mice and spontaneously obese mice reduced hyperinsulinemia and enhanced insulin sensitivity, suggesting that CA can improve obesity-related insulin resistance [31]. In mice exposed to high-fat milk, CA elevated insulin sensitivity and reduced insulin resistance [32]. Finally, when obese mice were exposed to a high-fat diet, the administration of CA-containing green coffee bean extract reduced the insulin resistance in a dose-dependent manner [33]. However, other clinical experiments have shown that the observed coffee-induced reduction of liver insulin resistance caused by short-term fructose overdose cannot be attributed to CA or caffeine, but to other unidentified active compounds [34].

### 2.4. Effect of CA on the Activity of Enzymes Involved in Glucose and Lipid Metabolism

The activity of glucose and lipid metabolism-associated enzymes and their regulation by natural products has become a research focus in the prevention and treatment of DM [35]. *In vitro*, CA modulates the activity of enzymes involved in glucose metabolism. Indeed, 100 *μ*g/mL CA competitively inhibited *α*-amylase, reducing its activity by 75%, similar to the inhibitory effect of acarbose [36], which is consistent with the results by Oboh et al. [37]. CA has also been shown to inhibit *α*-glucosidase activity, but this effect was far weaker than that of acarbose [38]. Other *in vitro* studies have shown that CA competitively inhibits glucose-6-phosphatase in the liver and reduces the hydrolysis of hepatic glycogen, thus contributing to the prevention and treatment of DM [39].

In a previous study, a 4-caffeoyl group was shown to be responsible for the observed inhibition by CA [40]. CA inhibited both isozymes of porcine pancreatic *α*-amylase (PPA), PPA-I, and PPA-II, suggesting that *α*-amylase inhibitors could be used to prevent and treat DM. CA can also modulate the activity of enzymes involved in lipid metabolism. Wenna et al. [41] showed that either a CA-containing extract of *Eucommia ulmoides* or pure CA reduced intestinal absorption and further conversion of lipid and cholesterol and also reduced liver cholesterol synthesis. However, in that study, the inhibition of pancreatic lipase activity was stronger for the *Eucommia ulmoides* extract than for the same concentration of control CA, suggesting that the extract may also contain other effective synergistic components. Lastly, in obese mice exposed to a high-fat diet, CA regulated lipid metabolism by inhibiting the activity of fatty acid synthase, HMG-CoA reductase, and cholesterol acyltransferase [42].

### 2.5. Effects of CA on DM Signal Transduction Pathways

Insulin mediates glucose metabolism in the body and exerts its biological activity after interaction with receptors. The signal is then transferred to the cell interior mainly via a tyrosine kinase pathway. Insulin signal transduction involves the insulin receptor substrate (IRS), phosphatidylinositol-3-kinase (PI3K) and serine/threonine kinase (Akt), and the glucose transporter (GLUT), which are the foci of current research on the molecular mechanism of insulin resistance [43].

CA is the main phenolic acid in *Sonchus oleraceus*, which improved insulin sensitivity in HepG2 cells [44]. It also reduced the decrease in IRS-1 expression caused by high insulin concentration, prevented inactivation of the PI3K/Akt pathway, and also prevented GLUT4 level reduction observed after high glucose exposure. These results are consistent with those of Liang et al. [32], where CA-treated mice previously exposed to high-fat milk showed an increase in GLUT-4 mRNA levels in skeletal muscle. However, other components in the phenolic acid extract from *Sonchus oleraceus* other than CA may be responsible for this effect. Similarly, whether the effect on the tyrosine kinase pathway is exerted by CA or by other components remains unknown.

In intestinal segments of rats exposed to a high-fat diet, Peng et al. [45] showed the suppression of GLUT2 downregulation after CA administration. Moreover, animal experiments showed that this downregulation may have been mediated by the activation of adenosine 5′-monophosphate-activated protein kinase (AMPK) facilitated by CA [46]. Indeed, these authors showed that CA promoted phosphorylation of AMPK and Akt to increase the transport of GLUT4 to plasma membranes, thus facilitating the transport of glucose. In fact, GLUT4 transportation could not be observed after the knockout of AMPKa1/2 or AMPK inhibition. CA was also shown to promote the expression and translocation of GLUT4, eventually inhibiting liver glucose production, but this inhibition disappeared after AMPK inhibition or knockout [23]. Nevertheless, caffeic acid, a metabolite of CA, rather than CA itself, may be ultimately responsible for the activation of AMPK in skeletal muscle to facilitate glucose transport [47].

### 2.6. Effects of CA on Oxidative Stress and Inflammatory Response

Oxidative stress and inflammatory response are key factors in the occurrence and development of type 2 DM [48–50]. These factors result in islet *β* cell injury [51, 52], accelerate insulin resistance [53, 54], and increase the development of DM-related complications [55, 56]. Therefore, the prevention and treatment of DM should benefit from alleviating oxidative stress and inflammatory response.

In DM model rats, the administration of CA reduced the content of lipid hydrogen peroxide and increased the content in nonenzymatic antioxidants in the blood such as glutathione (GSH), vitamin C, vitamin E, and ceruloplasmin [57], suggesting that CA protects against DM exposed to streptozotocin-nicotinamide-induced oxidative stress. In liver and kidney, CA reduced the levels of thiobarbituric acid reactive substances and hydroperoxide and enhanced the activity of superoxide dismutase (SOD), catalase (CAT), glutathione peroxidase (GSH-Px), and glutathione S-transferase (GST) [58]. In liver and white adipose tissues, CA inhibited protein and mRNA expression of F4/80 and CD68 and relieved inflammatory response [31]. In addition, CA had a protective effect on insulin secreting-IE (INS-1E) cells exposed to streptozotocin (STZ) [28]. In that study, CA promoted insulin secretion in INS-1E cells and increased GSH content and GSH-Px activity. Also, it reduced the production of reactive oxygen species (ROS) and the morphological changes of cells caused by STZ, thus protecting *β* cells.

## 3. Use of CA to Prevent and Treat DM-Related Complications

### 3.1. Effects of CA on Diabetic Nephropathy (DN)

DN is one of the most common microvascular complications of DM and also one of the main causes of death in DM patients [59]. There have been some attempts to use CA in the prevention and treatment of DN. In experimental DM rats, CA decreased kidney malondialdehyde (MDA) levels, increased SOD and GSH-Px activity, and reduced the expression of factors (IL-6, TNF-*α*, and IL-1*β*) related to oxidative stress and inflammatory response in the kidney [60]. In that study, pathological examination found that CA reduced glomerular hypertrophy and mesangial matrix expansion. Another animal experiment was consistent with these results, showing that CA enhanced SOD, GSH-Px, and CAT activity in the kidney, reduced the MDA levels, downregulated the expression of cyclooxygenase 2 (COX-2) protein, and reduced the proliferation and mesangial expansion of mesangial cells [61]. Thus, the above results suggest that CA may prevent and treat DN by relieving oxidative stress and inflammatory response in the kidney.

### 3.2. Effects of CA on Diabetic Retinopathy (DR)

DR is a microvascular complication of DM that is the main cause of vision impairment in the middle-aged and elderly people worldwide [62]. Thus, the role of CA in the prevention and treatment of DR has been the subject of intense research. Treatment of DM mice with honeysuckle extract, shown by high-efficiency liquid chromatography to mainly contain CA, suppressed STZ-induced retinal vascular proliferation and reduced content of vascular endothelial growth factor (VEGFs) in serum [63]. Also, in cellular and animal experiments, CA counteracts the effect of hypoxia-inducible factor 1-*α* and decreases VEGF expression during DR, thus improving retinal neovascularization. These results are consistent with retinal immunofluorescence staining of clusters of differentiation and histopathological observations [64]. In addition, in DM rats, CA improved the reduction in occludin, a tight junction protein and a component of the blood-retinal barrier, and inhibited the expression of VEGFs [65]. Overall, the above results show that CA can mitigate the effect of DR in the context of retinal vascular permeability.

### 3.3. Effects of CA on Diabetic Peripheral Neuropathy (DPN)

One of the most common chronic complications of DM is diabetic systemic disease, mainly manifested as peripheral neuropathy [66]. Thus, studies have been directed towards the effect of CA on diabetic neuropathy. In DM mice, CA improved the auditory function of the external auditory canal, alleviated the dysfunction of the central auditory pathway, contributed to the recovery of cochlear outer hair cell injury, prevented neuroma, and protected ear hair cells [67]. These effects are consistent with an ameliorating effect of CA on the auditory function. Also, using a mechanical claw pressure test, CA was effective to relief DM-induced neuropathic pain, possibly through reducing blood glucose level and alleviating oxidative stress [68].

## 4. Summary and Outlook

CA is a natural product obtainable from a variety of sources that has a wide pharmacological range. Compared with the existing hypoglycemic drugs, it has low toxicity or side effects. Because of its multisystem and multitarget pharmacological effects, CA may become a useful clinical drug in the treatment of the complex pathogenesis typical of DM and also its related complications. However, the present review shows that there are still many limitations in the application of CA for this purpose. First, despite the use of CA in the prevention and treatment of DM in different conditions, the mechanism of action and specific targets remain unclear. Second, the dosage of CA applied in DM needs to be confirmed by further evidences. Third, past efforts have only focused on DN, DR, and DPN, but these studies involve no diabetic cerebrovascular diseases and diabetic heart diseases. Fourth, there is a potential for the combined application of CA with Western hypoglycemic drugs or with other traditional Chinese medicines. These may bring reduced toxicity and enhanced efficacy to attack the most salient effect of DM, which is blood glucose. Finally, the development of CA as a new drug for the prevention and treatment of DM requires improvement in stability, solubility, and oral absolute bioavailability.
